# Formulation of Cannabidiol in Colloidal Lipid Carriers

**DOI:** 10.3390/molecules26051469

**Published:** 2021-03-08

**Authors:** Nadine Monika Francke, Frederic Schneider, Knut Baumann, Heike Bunjes

**Affiliations:** 1 Institute of Pharmaceutical Technology and Biopharmaceutics, Technische Universität Braunschweig, Mendelssohnstraße 1, 38106 Braunschweig, Germany; nadine.francke@outlook.com; 2 Institute of Medicinal and Pharmaceutical Chemistry, Technische Universität Braunschweig, Beethovenstraße 55, 38106 Braunschweig, Germany; frederic.schneider@tu-braunschweig.de (F.S.); k.baumann@tu-braunschweig.de (K.B.); 3 Center of Pharmaceutical Engineering (PVZ), Technische Universität Braunschweig, Franz-Liszt-Straße 35a, 38106 Braunschweig, Germany

**Keywords:** cannabidiol, lipid emulsion, colloidal lipid carriers, solid lipid nanoparticles, drug localization, emulsion stability

## Abstract

In this study, the general processability of cannabidiol (CBD) in colloidal lipid carriers was investigated. Due to its many pharmacological effects, the pharmaceutical use of this poorly water-soluble drug is currently under intensive research and colloidal lipid emulsions are a well-established formulation option for such lipophilic substances. To obtain a better understanding of the formulability of CBD in lipid emulsions, different aspects of CBD loading and its interaction with the emulsion droplets were investigated. Very high drug loads (>40% related to lipid content) could be achieved in emulsions of medium chain triglycerides, rapeseed oil, soybean oil and trimyristin. The maximum CBD load depended on the type of lipid matrix. CBD loading increased the particle size and the density of the lipid matrix. The loading capacity of a trimyristin emulsion for CBD was superior to that of a suspension of solid lipid nanoparticles based on trimyristin (69% vs. 30% related to the lipid matrix). In addition to its localization within the lipid core of the emulsion droplets, cannabidiol was associated with the droplet interface to a remarkable extent. According to a stress test, CBD destabilized the emulsions, with phospholipid-stabilized emulsions being more stable than poloxamer-stabilized ones. Furthermore, it was possible to produce emulsions with pure CBD as the dispersed phase, since CBD demonstrated such a pronounced supercooling tendency that it did not recrystallize, even if cooled to −60 °C.

## 1. Introduction

The pharmaceutical application of extracts and ingredients of cannabis plants have received increasing attention in recent years. Cannabidiol (CBD) is one of these promising ingredients isolated from cannabis. Unlike Δ^9^-tetrahydrocannabinol (THC), CBD has no undesired psychoactive side effects that would pose a risk of drug abuse. CBD is therefore of particular interest for pharmaceutical use [1]. Various pharmacological effects of CBD are already known or under investigation including antiepileptic, anti-inflammatory, antioxidative or neuroprotective activity [2]. Accordingly, there is a broad variety of potential therapeutic applications. A pharmaceutical product with CBD as the sole active ingredient (Epidyolex^®^/Epidiolex^®^) has already been approved for the treatment of some severe types of epilepsy—e.g., Lennox–Gastaux syndrome or Dravet syndrome [3,4,5].

Besides further physicochemical properties, listed in Table 1, CBD is a lipophilic substance with a log P of 6.1 and is poorly soluble in water (0.01 mg/mL) [6]. Its low aqueous solubility poses a severe formulation challenge. Moreover, CBD has a poor bioavailability when administered orally [2]. Oily solutions for oral administration are currently the most commonly used formulations of CBD [3]. In order to increase oral bioavailability, CBD has already been formulated in dispersed lipid systems such as self-nanoemulsifying drug delivery systems (SNEDDS) [7,8]. Colloidal lipid formulations with adequate compositions and particle sizes would also be promising formulation options for parenteral administration of CBD [9]. An injectable formulation might be of interest to circumvent the limited oral bioavailability [2]. Considering the wide range of potential applications, a formulation that enables the use in patients with severe swallowing difficulties may also be required. Pharmacological research would also profit from a parenteral formulation, as it enables intravenous administration as reference in clinical studies and animal experiments [9].

This study investigated the processability of CBD in colloidal lipid emulsions and suspensions and the influence of CBD loading on the physicochemical properties of emulsions.

Different oils and emulsifiers can be used for the preparation of colloidal lipid emulsions. The selection of an appropriate oil can be essential for the achievable drug load [10]. Furthermore, it has already been demonstrated that the achievable drug load may depend on the physical state of the particles and may, depending on the drug, be higher in solid or liquid particles [11]. Supercooling, a special thermal behavior of triglycerides, allows comparison of equally composed colloidal emulsions and suspensions under favorable circumstances (at room temperature).

A possibility for gentle drug loading is the passive loading procedure [12,13,14]. Here, a drug-free emulsion is prepared and then incubated with the drug. This loading technique ensures that the active ingredient is not exposed to the thermal stress or shear forces that occur during high-pressure homogenization.

Drug molecules may be localized at different sites of the emulsion system. In addition to the aqueous phase and the lipid matrix of the emulsion droplets, drug localization is also possible at the droplet interface or in other colloidal structures (e.g., micelles) formed by an excess of emulsifier in the aqueous phase. To investigate localization of CBD with special regard to its distribution between droplet core and interface, the dependence of the achievable drug load on the available droplet interfacial area and thus of the mean droplet size of the emulsion, can be used [10,11].

Colloidal lipid emulsions are thermodynamically unstable systems, stabilized by emulsifier molecules situated in the droplet interface. The selected emulsifier as well as drug localization in the interfacial area can influence the stability of the emulsion [15,16]. Therefore, the emulsion stability after CBD loading was investigated by a self-developed shaking test [16].

## 2. Material and Methods

### 2.1. Materials

Cannabidiol was obtained from THC Pharm (Frankfurt, Germany). Tetrahydrofuran of high-performance liquid chromatography (HPLC) quality for UV spectroscopy was purchased from Sigma-Aldrich (Steinheim, Germany). Tetrahydrofuran of Ultra LC–MS grade and acetonitrile of LC–MS grade for HPLC measurements were ordered from Carl Roth (Karlsruhe, Germany), as well as sodium azide. Refined soybean oil and refined rapeseed oil were obtained from Caelo (Hilden, Germany). Lipofundin^®^ MCT/LCT 10% (referred to as Lipofundin^®^ in the following and composed of 5% soybean oil, 5% medium chain triglycerides, 2.5% glycerol, 0.8% egg lecithin and unspecified quantities of α-tocopherol and sodium oleate [18,19]) was obtained as commercial colloidal lipid emulsion from B.Braun (Melsungen, Germany). Poloxamer 188 (Kolliphor^®^ P 188) and poloxamer 407 (Kolliphor^®^ P 407) were kind gifts from BASF (Ludwigshafen, Germany) and trimyristin (Dynasan^®^ 114) and medium chain triglycerides (Miglyol^®^ 812 N) from IOI Oleo (Witten, Germany), respectively.

### 2.2. Preparation of Colloidal Lipid Emulsions

All colloidal lipid emulsions except the SB-P188-230 nm emulsion for localization experiments were produced by high-pressure homogenization in a Microfluidizer M-110 P with an interaction chamber type F12Y (Microfluidics, Newton, MA, USA). The compositions are given in Table 2. The aqueous (sodium azide, emulsifier, bidistilled water) and lipophilic phases were weighed separately and the aqueous phase was stirred until dissolution of the ingredients. The phases were unified and dispersed for 5 min with a speed of 11,000 rpm by an IKA T 25 Ultra Turrax digital, equipped with a S 25 N-10 G dispersing tool (IKA, Staufen, Germany). The resulting dispersion was high-pressure homogenized approximately 10 times through the microfluidizer (for the applied pressures, see Table 2). All emulsions were filtrated immediately after homogenization through 0.45 µm PVDF filters (Carl Roth). In case of the trimyristin emulsion, the two phases were first heated above the melting temperature of trimyristin to a temperature of 85 °C. The complete production of the trimyristin emulsion was performed at this temperature as described above. After cooling to room temperature and during storage at 20 °C, the trimyristin droplets remained in the state of a supercooled liquid melt [20]. To obtain the trimyristin suspension, 10 mL of the diluted trimyristin emulsion TM-P188-120 nm was placed in a refrigerator for three days, leading to crystallization of the trimyristin (for dilution, see Section 2.6).

The SB-P188-230 nm emulsion was prepared by premix membrane emulsification in a self-constructed membrane extruder equipped with a high-pressure syringe pump (Cetoni, Korbußen, Germany) [21]. The composition is also given in Table 2. The premix was produced with an Ultra Turrax as described above. Afterwards, the premix was automatically processed 21 times through a 0.2 µm polyester filter membrane (Pieper Filter, Bad Zwischenahn, Germany) with a flow rate of 0.8 mL/s using a 25 mL stainless steel syringe. Finally, the emulsion was filtrated through 1–2 µm glass fiber filters (Carl Roth). Two comparable emulsions were produced and unified after particle size control to achieve a larger sample volume.

The designation of emulsions used in the text includes the type of lipid, the applied emulsifier and the approximate particle size (see Table 2).

The emulsions of pure melted CBD (without lipid) as the dispersed phase were prepared by a dual centrifugation technique in a Zentrimix 380R (Hettich, Tuttlingen, Germany) centrifuge. The centrifuge was modified by the manufacturer with a heating option, resulting in a usable temperature range of −20 to 90 °C. Yttrium-stabilized zirconium oxide (ZrO2) beads Type ZY-P Pharma (Sigmund Lindner, Warmensteinach, Germany) with a density of 6.05 kg/dm^3^ and a particle size of 0.5 to 0.7 mm were used as homogenization media in a filling ratio of φ = 0.15 [22]. The emulsifier was dissolved in bidistilled water under stirring to prepare the aqueous phase. Afterwards, the homogenization beads, CBD and the aqueous phase were added directly into disposable 2 mL centrifugation tubes. The resulting formulation contained 5% CBD and 12% emulsifier (poloxamer 188 or poloxamer 407). The centrifuge was preheated to 60 °C for at least 30 min in order to achieve a constant temperature in the centrifugation chamber. The tubes were placed in the sample holder and processed for 10 min at 60 °C and 2350 rpm. Each emulsion of CBD was prepared twice.

### 2.3. Particle Size Determination

The intensity weighted particle sizes expressed as z-average diameters and polydispersity indices (PdIs) were determined with a ZetaSizer Nano Series ZS (Malvern Panalytical, Kassel, Germany) at 25 °C and an angle of 173°. To obtain an appropriate scattering intensity, samples were diluted with bidistilled water to an attenuator between 6 and 8. After an equilibration time of 300 s, measurements were performed in triplicate with a measurement time of 300 s each.

Laser diffraction was applied to determine the particle size distribution. Measurements were performed in a HORIBA LA-960 (HORIBA, Oberursel, Germany), equipped with a manual fraction cell, after dilution in bidistilled water to an appropriate transmission of less than 90%. Every sample was prepared once and measured in triplicate. The evaluation was based on the Mie theory and calculated as the volume distribution of the emulsion droplets. Calculations for lipid-containing systems were based on a refractive index of 1.46 for the dispersed phase (imaginary part: 0.01) and 1.33 for the aqueous phase. The refractive index of 1.46 is the average of the refractive indices of the applied oils (soybean oil: 1.475 [23], medium chain triglycerides: 1.446 [24], rapeseed oil: 1.473 [25] and trimyristin: 1.443 [26]).

### 2.4. Lipid Quantification

During emulsion production, the emulsion may be slightly diluted by process water remaining in the emulsification device, which results in a slightly lower lipid content in the produced emulsion. Since two different manufacturing processes were used to produce the three emulsions compared in the localization experiment, it is to be expected that the loss of lipids will be slightly different. The lipid content of the emulsions was determined by high-performance liquid chromatography (HPLC) after their preparation, to compare the loading of these emulsions more accurately. This measurement was performed in a Dionex UltiMate 3000 HPLC System (Pump LPG-3400SD and sampler WPS-3000TSL) (Thermo Fisher Scientific, Dreieich, Germany). A mixture of tetrahydrofuran and acetonitrile (25:75) was applied as eluent with a flow rate of 0.3 mL/min and for sample preparation the emulsions were dissolved and diluted in a mixture of tetrahydrofuran and acetonitrile (50:50). The Hypersil Gold C18 Column 2.1 × 150 mm with a particle size of 1.9 µm (Thermo Fisher Scientific, Dreieich, Germany) was temperature-controlled to 25 °C by the column compartment (column oven TCC-3000SD). A Corona Veo Charged Aerosol Detector was used at a nebulization temperature of 50 °C for peak detection. For data recording, the following settings were applied: data collection rate: 10; filter: 1.0; power function: 1.0.

### 2.5. Determination of Emulsifier in the Aqueous Phase

For the determination of the emulsifier content in the aqueous phase of the emulsion, the aqueous phase was separated by a self-developed centrifugation technique and analyzed refractometrically afterwards. Vivaspin^®^ 6 centrifugal concentrators with a cut-off of 300 kDa (Sartorius, Göttingen, Germany) were applied for separation. All centrifugation steps were performed at 22 °C with 500 *g* in an Allegra 64R centrifuge (Beckman Coulter, Krefeld, Germany). Before centrifugation of the emulsions, the Vivaspins^®^ were prepared in three washing steps. In each step, 5 mL of bidistilled water was centrifuged for 10 min to remove production-related residues of glycerol and sodium azide from the filter membrane, as these would interfere with the subsequent analytical procedure. To avoid dilution effects, a 10 min centrifugation step in dry condition was added to remove water residues from the membrane. Afterwards, 5 mL of emulsion was filled into the Vivaspins^®^ and centrifuged for 60 min, to achieve the separated aqueous phase as filtrate.

The emulsifier content in the filtrate was determined refractometrically in an Abbemat-WR refractometer (Anton Paar, Ostfildern-Scharnhausen, Germany). The refractive index of each filtrate was measured three times at a temperature of 25 °C. The evaluation was performed based on calibration lines.

### 2.6. Passive Drug Loading

A previously described drug loading procedure was applied for the CBD loading of the carriers [10,12]. The respective drug-free carrier or oil was incubated with bulk CBD in glass vials (UV-protected and flushed with N_2_) under shaking (horizontal shaker IKA Vibrax MS3 digital, IKA, Staufen, Germany) with a rotation speed of approximately 300 rpm at 20 °C. The selected vial sizes led to a filling level of between 50% and 60% to ensure good homogenization during the shaking process. After loading, an excess of drug was removed by filtration through 0.45 µm PVDF filters (Carl Roth). For the filtration of the emulsions in the localization experiment (SB-P188-70 nm, SB-P188-150 nm and SB-P188-230 nm) and of loaded Lipofundin^®^, 1–2 µm glass fiber filters (Carl Roth) were used. In a control experiment, it was confirmed that CBD did not adsorb on any of the applied filter types. Table 3 presents the loading times, initially added CBD concentrations and sample quantities for all passive drug loading experiments.

Before all drug loading procedures, except those for the stability tests, the emulsions were diluted with an emulsifier solution to 2% lipid. In most cases the concentration of the emulsifier in these solutions corresponded to the emulsifier content in the aqueous phase of the respective emulsion (Table 4). The emulsions used for the localization experiments were diluted with a 1.6% poloxamer 188 solution, which corresponds to the aqueous phase of the medium-sized emulsion SB-P188-150 nm. To obtain the trimyristin suspension, the diluted trimyristin emulsion TM-P188-120 nm was placed in a refrigerator (see Section 2.2).

In the investigation of the drug loading kinetics, each time point was analyzed by using separately loaded vials. CBD was weighed into the 2 mL vials and 1 mL of the diluted emulsion was added.

### 2.7. Drug Quantification

The CBD concentrations were determined by UV–Vis spectroscopy in a Specord 40 spectrometer (Analytik Jena, Jena, Germany). A calibration curve was recorded and the samples were diluted with tetrahydrofuran/water 9/1 (*v*/*v*) to an absorption for which a linear correlation was confirmed. The measurements were performed at a wavelength of 212 nm. The solvent was used as baseline and the absorption of the respective unloaded emulsion was subtracted as a blank value from that of the sample.

### 2.8. Shaking Test

Eppendorf tubes^®^ (1.5 mL) were filled with 300 µL of emulsion and placed in PTFE beakers, perforated for Eppendorf tubes^®^. The beakers were shaken with 25 Hz for different periods of time in a Retsch MM301 oscillating mill (Retsch, Haan, Germany). For each investigated time point, three separately prepared tubes were filled and shaken (e.g., nine tubes for three investigated periods of time). A sample was defined as unstable when laser diffraction analysis indicated that two of the three samples had lost their monomodal size distributions.

### 2.9. Differential Scanning Calorimetry

The thermal behavior of CBD bulk material and emulsions was analyzed with a Mettler Toledo DSC 1 equipped with an FRS 5 sensor. The oven was flushed with nitrogen. The samples were weighed in aluminum crucibles and sealed. The evaluation was performed against the reference temperature and signals were normalized to the sample weight.

CBD bulk material was investigated with the following temperature-controlled program: 1. heating from 0 to 85 °C (heating rate: 5K/min); 2. cooling from 85 to −60 °C (cooling rate: 2.5 K/min); 3. heating from −60 to 85 °C (heating rate: 5 K/min).

The liquid state of the droplets in emulsions of pure CBD was investigated by heating of the samples from 20 to 100 °C with 10 K/min.

### 2.10. Density Measurements

To prepare the samples for density measurements of oily solutions with 2.5% or 5% CBD (related to the lipid), the respective amount of drug was dissolved in 3 g soybean oil. Afterwards, the experimental density determinations were performed in a DMA 1001 (Anton Paar) by evaluating the oscillation characteristics. The instrument was calibrated by air–water calibration and before each measurement this calibration was checked with a water test. Each sample was filled once into the measuring capillary and measured three times.

### 2.11. Computer Simulations

To simulate the density of CBD in soybean oil, molecular dynamics simulations were performed with the GROMACS software package version 2018.04 [27]. The GROMOS96 54A7 united atom forcefield [28] was used and missing parameters to generate the desired topologies were computed using the ATB Server [29]. To represent the soybean oil, only the two major triglycerides (triolein and trilinolein) were considered and if available in the ATB database, different starting conformations were used. For CBD, a new entry was created (ATB ID: 366608). The number of molecules was calculated in such a way as to reach the desired composition of each concentration and the selected ratio for soybean oil (triolein/trilinolein: 30.45/69.55%) in a cubic simulation box of approximately 8 nm^3^. The starting box of the simulation was prepared with PACKMOL by using a larger initial box size in order to be able to place each molecule in a consistent distance [30]. The conformation composition of soybean oil was selected randomly. For all simulations, the Langevin dynamics integrator with a timestep of 1 fs together with the reaction field for the nonbonded coulombic interaction was used [31]. The simulation was subdivided into two parts. First, a three-part equilibration was carried out to achieve a starting point for data collection. Afterwards the density was collected for a period of 100 ns in a constant temperature (293 K) and pressure (1 bar) simulation. The temperature was controlled by the integrator and the pressure was controlled by the Berendsen barostat (during equilibration) as well as the Parrinello–Rahman barostat [32,33].

## 3. Results and Discussion

### 3.1. Kinetics of Drug Loading

**Figure 1:**
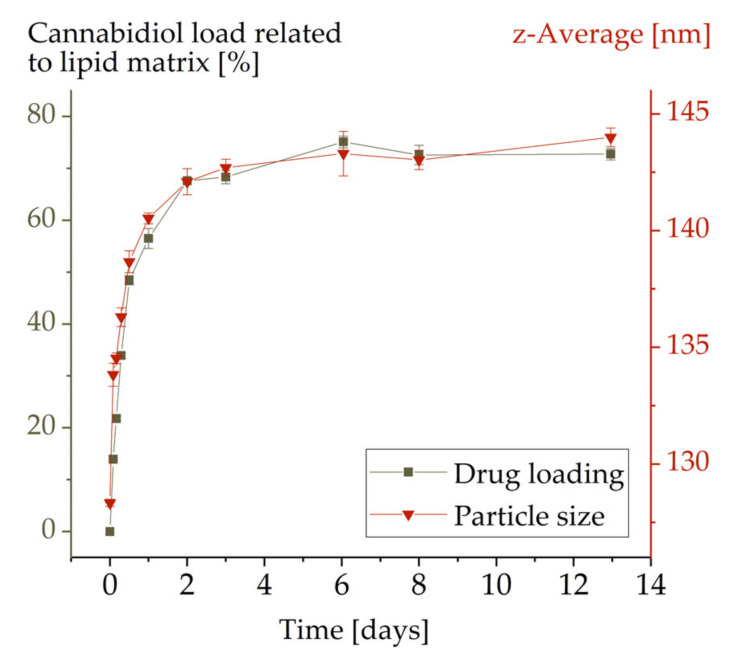
Kinetics of cannabidiol loading in emulsion MCT-P188-120 nm. Error bars represent the standard deviations but are mostly not visible.

In order to determine a suitable time period to achieve a maximum drug load in the emulsion samples, the kinetics of the loading process of emulsion MCT-P188-120 nm were studied. Data were collected until the samples were saturated with CBD (Figure 1).

For MCT-P188-120 nm, the drug load increased over time up to a CBD content of 73% in relation to the lipid content. The maximum drug load was reached after approximately 5 days. Therefore, in order to ensure complete loading, a period of at least 7 days was specified for all other drug loading procedures. The observed loading capacity of 73% (related to the lipid) is remarkably high when compared to that for other drugs, which is mostly lower than 10%–20% [10,11,12]. By means of passive drug loading, a similarly high drug loading could previously only be achieved for propofol [11].

Concurrent with the drug loading, there was an increase in particle size confirming that CBD was truly loaded to the emulsion droplets and was not only being dissolved in the aqueous phase (Figure 1). The increase in particle diameter (about 13%) was, however, surprisingly low considering the high incorporation of CBD. This issue will be considered in more detail in Section 3.2 and Section 3.3.

As a further control of drug loading to the particles, the solubility of CBD in a 1.6% poloxamer 188 solution was determined (poloxamer concentration corresponds to that in the aqueous phase of the medium-sized emulsion SB-P188-150 nm). The solubility of CBD in this solution was so low that it was not detectable by the applied analytical method. This also confirmed that the CBD was indeed located in or on the emulsion droplet and not in the aqueous phase.

### 3.2. Influence of the Type of Lipid Matrix

Since the drug loading capacity of emulsions may strongly depend on the lipid used [10], this dependence was also investigated for CBD. The loading capacity was determined for poloxamer 188-stabilized emulsions based on different oils (medium chain triglycerides (MCT-P188-120 nm), trimyristin (TM-P188-120 nm), soybean oil (SB-P188-120 nm), and rapeseed oil (RS-P188-120 nm)). All emulsions had similar particle sizes of 120 ± 4 nm, so that the available interfacial areas of their emulsion droplets were comparable (Table 4).

**Figure 2:**
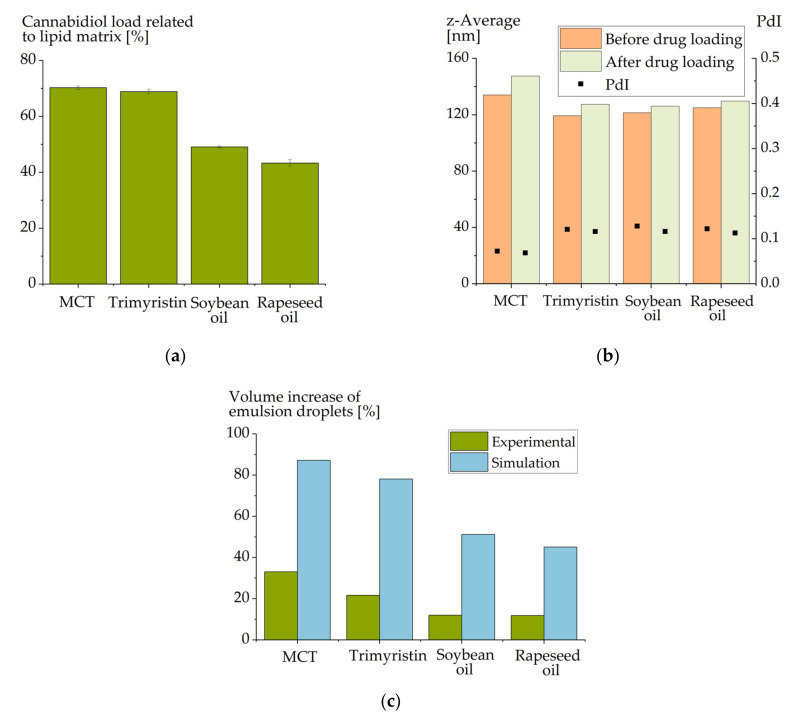
(**a**) Achievable cannabidiol load (mass percent) in emulsions composed of different matrix lipids (emulsions MCT-P188-120 nm, TM-P188-120 nm, SB-P188-120 nm and RS-P188-120 nm). (**b**) Particle sizes of these emulsions before and after cannabidiol loading. (**c**) Experimentally observed increase in the volumes of the emulsion droplets as compared to a simulated volume increase based on the molecular volume of the loaded cannabidiol molecules. The molecular volume was based on the van der Waals surface which was computed with MOE Version 2018.01.01.

The achievable CBD load in mass percent exhibited a clear dependence on the selected lipid (Figure 2a). In general, a surprisingly high load of more than 40% CBD related to the lipid matrix was observed for all emulsions. The emulsions based on medium chain triglycerides and trimyristin even obtained a CBD load of about 70%, whereas the loads for soybean oil (49%) and rapeseed oil (43%) were lower.

The results corresponded very well with the already described higher solubility of poorly water-soluble drugs in medium chain compared to long-chain triglycerides [34,35]. Due to their higher polarity, esterified short-chain fatty acids interact more intensively with the induced drug molecules. The highest CBD load was achieved in MCT, which is mainly composed of saturated C8 and C10 fatty acids (Table 5). This observation is consistent with investigations on other drugs, in which drug loading of MCT led to higher or at least comparable drug concentrations compared to drug loading of trimyristin and rapeseed oil [10]. Like CBD, most drugs reached a higher load in trimyristin compared to rapeseed oil.

The comparably high CBD load in MCT and trimyristin can be discussed from two different points of view. On the one hand, due to the higher polarity of MCT, a higher drug load in MCT than in trimyristin would have been expected. A reason for the comparable drug load in trimyristin may be that the drug load achieved in trimyristin was already very high and due to space requirements in the lipid matrix could not be increased further by a higher polarity of the matrix lipid. On the other hand, the considerably higher drug load of trimyristin, compared to soybean and rapeseed oil, can be discussed. One explanation may be the slightly shorter-chain length of trimyristin and, thus, its higher polarity. Furthermore, soybean oil and rapeseed oil contain mainly unsaturated fatty acid residues. Double bonds strongly restrict the mobility of fatty acids and thus may hamper the incorporation of CBD compared to the saturated chains of trimyristin.

A molar relationship between the lipid and the available drug load has been described in previous studies [36,37]. In these studies, the molar concentration of the ester bonds rather than the chain length itself was proposed to be important for the drug loading capacity of oils. The calculated molar ratio between the molar CBD loading and the average molar weight of the respective fatty acid was between 0.35 (MCT) and 0.50 (trimyristin). The results for soybean oil (0.44) and rapeseed oil (0.39) were between these two values. Based on these results, no conclusions can be drawn as to whether the CBD concentration in MCT or trimyristin is unexpectedly high. However, from a molar perspective, the difference in the drug loading capacity of the emulsions composed of different oils is not as high as when regarding the mass-related drug loadings.

Again, a slight increase in the particle size was observed in all emulsions upon CBD loading (Figure 2c). In order to estimate to which extent an increase in particle size would have to be expected, a very coarse estimation of the space requirement of the loaded cannabidiol molecules was made. The Van der Waals volume of cannabidiol was calculated and, based on this calculation, the approximate space requirement of the introduced number of cannabidiol molecules was estimated (Figure 2c). Obviously, this estimation is very coarse since molecular interactions of the CBD molecules with the matrix were completely ignored. Nevertheless, considering these results, a much larger particle size increase would have been expected in the experiments.

### 3.3. Effect of Drug Loading on Oil Density

As a possible cause for a smaller increase in particle size than the coarsely estimated one, the influence of CBD on the density of the oil was investigated. Since it was not possible to (experimentally) investigate the density in the range of CBD saturation owing to the cost of the considerable sample quantities required, this was carried out using molecular dynamics simulations. For low CBD concentrations, the simulation results were compared to experimental data.

The experimentally determined density of pure soybean oil was 919.7 kg/m^3^ (20 °C), which is close to literature data (919.3 kg/m^3^ (23.9 °C)) [42]. Upon addition of CBD, experimental as well as simulated data indicated an increase in density due to the addition of CBD. With a mass fraction of 2.5% CBD, an experimental increase in density of 2.25 kg/m^3^ was observed while the estimated increase based on the molecular dynamics simulation was 2.26 kg/m^3^. A higher mass fraction of 5% CBD resulted in an experimental increase of 4.56 kg/m^3^ and a simulated increase of 4.45 kg/m^3^. This demonstrates that the employed simulation protocol is suitable in this CBD concentration range.

In simulations, the density also increased with higher CBD concentrations and reached a density of 963.5 kg/m^3^ with equal mass proportions of soybean oil and CBD (100% CBD).

To investigate the extent to which the density was influenced when further CBD was added to an oil already containing CBD, the relative increase in the density normalized to the CBD concentration was calculated as follows:

rel. Increase in Density = (Density (x%) − Density (0%))/(x%)(1)

**Figure 3:**
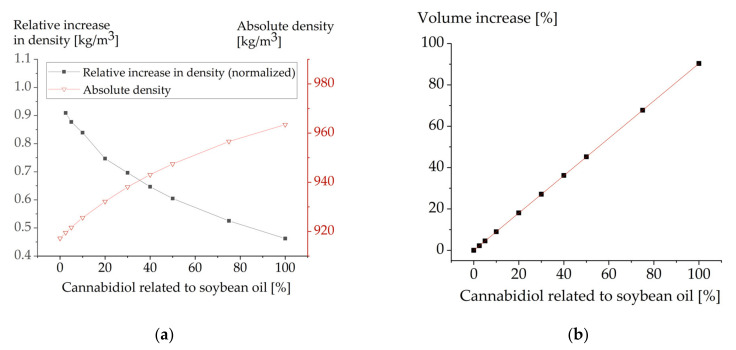
(**a**) Effect of cannabidiol on the density of soybean oil, demonstrated as density and calculation of the relative increase in density normalized to the cannabidiol concentration. (**b**) Simulated volume increase of soybean oil with increasing concentrations of cannabidiol.

In the nominator, the difference of the density of soybean oil with the corresponding CBD content and that of the CBD-free soybean oil was calculated. Subsequently, to be able to compare the resulting values, they were normalized to the CBD content (x%). The percentage of CBD that was used in these calculations reflects the mass that was added to soybean oil. The composition at “100% CBD” was thus 100% soybean oil + 100% CBD (Figure 3).

Figure 3a illustrates that the effect of additionally introduced cannabidiol molecules on the density of the matrix decreases with increasing CBD content. This may be explained by the increasing probability that the newly introduced cannabidiol molecules can interact not only with the lipid molecules but also with one another.

Nevertheless, the increase in density alone cannot explain the small increase in particle size (in experiments). The particle size increase of SB-P188-120 nm observed upon passive loading with 49% CBD (m% related to soybean oil) corresponds to an increase in volume of 12% (Figure 2c). The coarse calculations based on the van der Waals volume as approximated by MOE predict an increase of 51%. When the volume increase was calculated based on the computed density, the predicted value was 45% and thus lower (Figure 3b). Yet, it still overestimates the volume increase by a large margin. Hence, additional factors will influence volume increase. On the one hand, the introduction of cannabidiol may result in a better packaging of the molecules in the lipid matrix and thus the volume requirement would be lower. On the other hand, emulsions are far more complex systems than pure drug–lipid compositions. In addition to the lipid, emulsifier molecules and an interface composed of them is of utmost importance. Due to their complexity, emulsifiers and their behavior cannot be included in this type of simulation and thus represent an unknown that may—at least in part—explain the overestimation of volume increase.

### 3.4. Drug Localization

Due to their small particle size, colloidal lipid emulsions have a large available interfacial area and it is already known that this interfacial area can be used for drug localization [10,11]. The interaction of CBD and the interface of the emulsion droplets is another interesting aspect. Thus, a potential further increase in the drug loading capacity by a decrease in the particle size of the emulsion was also evaluated.

Three soybean oil emulsions of different mean droplet sizes but the same compositions were used in this experiment (SB-P188-70 nm, SB-P188-150 nm and SB-P188-230 nm). The emulsions were loaded up to saturation with CBD. The achievable drug load was related to the lipid content in the emulsions that had been analyzed after emulsion production. These emulsions differed only in their particle sizes and, thus, in their available interfacial areas of the emulsion droplets. More interface is available in smaller-sized emulsions than in larger-sized ones.

**Figure 4:**
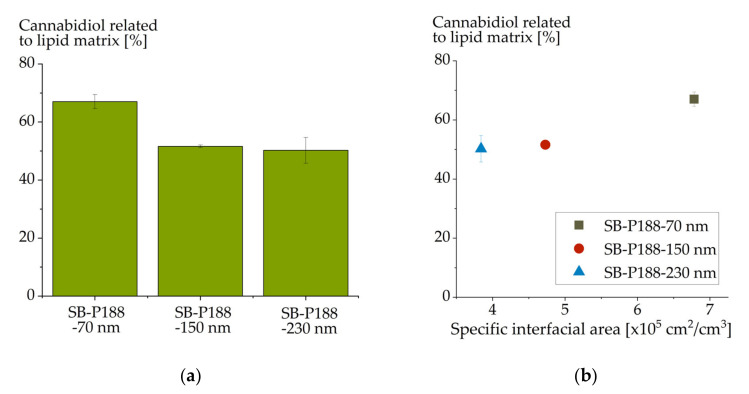
Cannabidiol load (mass percent) of differently sized colloidal soybean oil emulsions (**a**); (**b**) illustrates the drug load in dependence on specific interfacial area of the emulsion droplets.

CBD load considerably increased with decreasing particle size (Figure 4a). When plotting the achievable CBD concentration against the interfacial area of the emulsion droplets (Figure 4b), an increase in the drug loading capacity with available interfacial area was obvious. CBD thus appears to also have a certain tendency for surface localization in colloidal lipid emulsions. Since a particle size reduction from 150 to 70 nm increased the drug loading capacity from 52% CBD to 67% (related to the lipid matrix), it is advantageous to choose a small particle size in formulation development.

However, a high loading capacity was achieved even with larger droplet sizes. Thus, a large fraction of the CBD still localized in the lipid matrix. Based on the already described assumption that interface-localized drugs induce an exponential correlation between their drug loading capacity and the available interfacial area [15], an exponential fit of the CBD data was performed (see Supplementary Materials). The extrapolated data fit to a theoretical interfacial area of 0 m^2^/m^3^ resulted in 495 mg CBD/g lipid. Since the CBD loaded onto the interface was excluded by this evaluation, this concentration should represent the CBD concentration in the lipid matrix. Drug loadings above this concentration represent the CBD concentration in the droplet interface. The number of drug molecules per interfacial area of the emulsion droplets increased with decreasing particle size from 0.06 (SB-P188-230 nm) to 0.14 (SB-P188-150 nm) and 0.82 pmol/cm^2^ (SB-P188-70 nm). An equal effect was observed for other drugs and was attributed to the increasing curvature of the surface with decreasing particle size [15].

### 3.5. Influence of the Physical State of the Lipid Matrix

Since the drug loading capacity of CBD benefits from an increase in the interfacial area of the droplets, a colloidal triglyceride suspension may be a suitable formulation option. The particle shape usually changes from spherical in emulsions to platelet-like in suspensions and, thus, results in an increase in the available interfacial area [43,44].

Triglycerides exhibit a special thermal behavior that can be used to investigate colloidal lipid emulsions and suspensions containing the same lipid. Trimyristin is a solid, crystalline substance at room temperature in the bulk (melting point: 57 °C [17]), but it forms a supercooled melt after processing into the colloidal state. After melt homogenization, trimyristin does not recrystallize at room temperature and the emulsion droplets remain in the liquid state [17,20]. However, when the emulsion is cooled below a critical temperature (e.g., in a refrigerator), the lipid recrystallizes and the formulation is converted into a suspension of triglyceride nanoparticles [20]. This effect was exploited in order to compare the achievable CBD loading in an emulsion and a suspension. Using the trimyristin emulsion TM-P188-120 nm, CBD was loaded to two corresponding trimyristin formulations containing lipid particles in the liquid and crystallized state.

**Figure 5:**
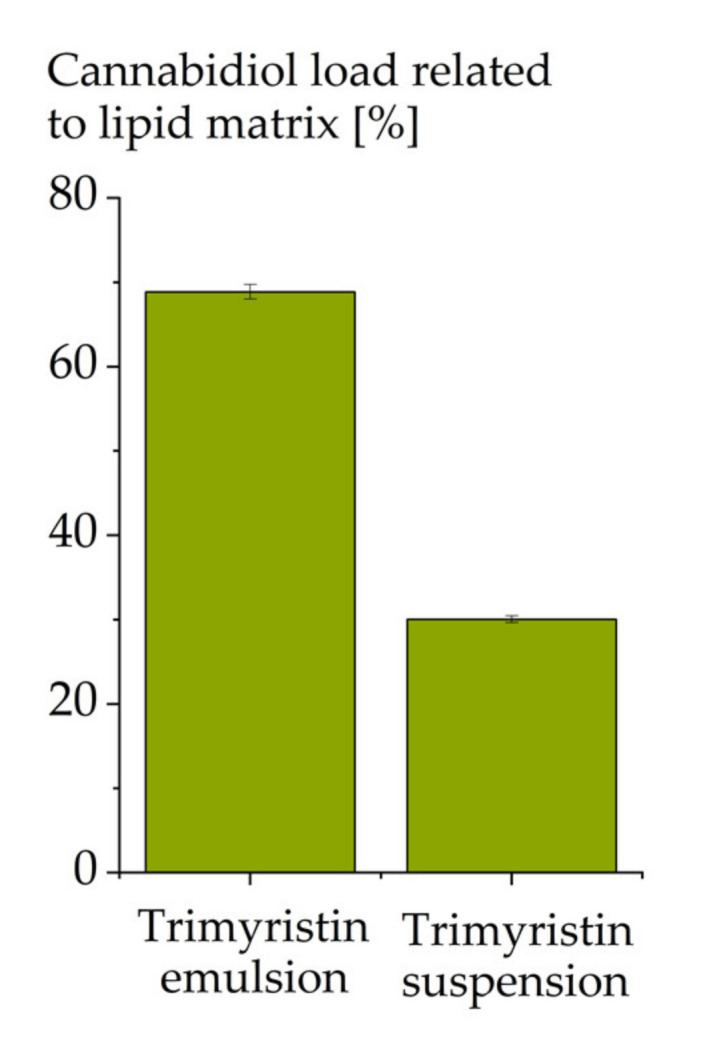
Cannabidiol load (mass percent) in a trimyristin emulsion and suspension of equal composition (from TM-P188-120 nm).

The achievable drug load decreased substantially from 69% in the trimyristin emulsion to 30% in the corresponding suspension (Figure 5). This correlates well with previous observations, where for most substances a lower drug load was achieved in suspensions compared to emulsions [11,12,45]. This may be explained by the reduced space available for the drug in the crystalline lipid matrix compared to the liquid lipid of the emulsion particles. After incorporation into an emulsion, the drug may be displaced to the surface or into the aqueous phase upon crystallization of the droplets [46,47]. Furthermore, upon passive drug loading, the drug can be expected to mainly be loaded onto the droplet interface, because it may be difficult for the drug to intrude into the crystalline structure of the lipid matrix. For CBD, a remarkably high drug concentration of 30% could still be loaded to the trimyristin suspension particles. This confirms the assumption that CBD partly localizes in the interfacial area instead of a pure matrix localization. Nevertheless, the increased interfacial area of the suspension particles cannot compensate the limited space in the lipid matrix caused by its crystalline state.

### 3.6. Influence of Drug Load on Emulsion Stability

**Figure 6:**
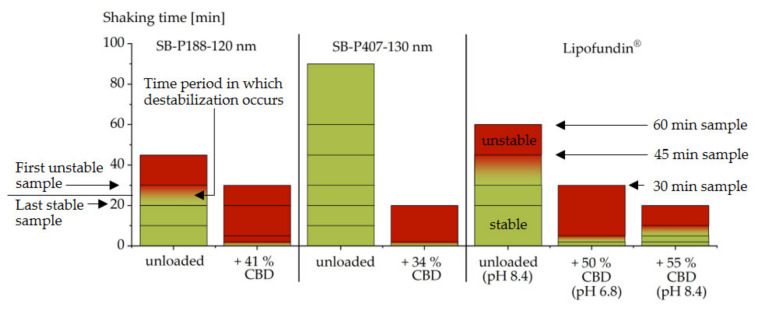
Stability of soybean oil emulsions stabilized with poloxamer 188 (P188) or poloxamer 407 (P407), respectively, and Lipofundin^®^ (composed of phospholipids, medium chain triglycerides and soybean oil) before and after loading with cannabidiol (CBD). Shaking periods that did not induce instability are shown in green, intervals of unstable samples in red; the first destabilizing interval is marked with a color gradient. Investigated samples points are included as lines.

Sufficient stability of drug-loaded emulsions is essential for their practical application. Due to the high drug load and the general thermodynamic instability of colloidal lipid emulsions, a potential influence of the CBD loading on the stability of the emulsion needs to be considered. To examine the stability, a self-developed accelerated shaking test was applied [15,16]. In the first step, the influence of CBD was investigated on the emulsion SB-P188-120 nm. Upon shaking, the unloaded emulsion became unstable between 20 and 30 min. The CBD loading of 41% led to a substantial destabilization of the emulsion, resulting in instability already at the first examination point (2 min) (Figure 6). Although a change to poloxamer 407 (emulsion SB-P407-130 nm) resulted in a substantially more stable unloaded emulsion, the same destabilization time of less than 2 min was observed after drug loading (34% CBD).

A destabilizing effect of drugs on emulsions sterically stabilized with poloxamer has been observed previously for other undissociable, surface-localizing drugs [15]. The results obtained with CBD are thus in good agreement with previous results. In terms of stability, the use of phospholipids as emulsifiers has shown advantages for formulations of drugs with these properties [16]. To test this approach for CBD, the commercial, phospholipid-stabilized emulsion Lipofundin^®^ (lipid phase: soybean oil + medium chain triglycerides) was included in the loading experiments. The unloaded Lipofundin^®^ had a z-average diameter of 212 nm (PdI 0.10), a pH of 8.4 and destabilized between 30 and 45 min upon shaking. For this emulsion, CBD loading (50% CBD) led to a substantial destabilization as well (Figure 6). The time required for destabilization was, however, between 2 and 5 min, which means that the CBD-loaded Lipofundin^®^ was slightly more stable than the poloxamer-stabilized emulsions with CBD. CBD loading caused a decrease in the pH from 8.4 to 6.8 in Lipofundin^®^. In a subsequent experiment, Lipofundin^®^ was loaded with CBD (55% CBD) and its pH was afterwards increased to the pH of the unloaded emulsion (pH 8.4) to compensate for the already known destabilizing effect of decreasing pH on the stability of phospholipid-stabilized emulsions [16,48]. This resulted in a higher stability of the emulsion (Figure 6). Although the CBD-loaded emulsion was still less stable than the drug-free emulsion, the achieved destabilizing interval (5 to 10 min) was consistent with that of the commercially available emulsion Propofol-Lipuro^®^, whose stability was investigated in a separate study with the same method [16]. When adequate formulation strategies are used, it appears feasible that even high concentrations of CBD can be formulated into emulsions with sufficient stability for practical application. A reason for the remaining destabilizing effect of CBD may be its extraordinarily high loading, which was by far not achieved with most other drugs [10,11,12,13].

### 3.7. Emulsions of Supercooled Cannabidiol

**Figure 7:**
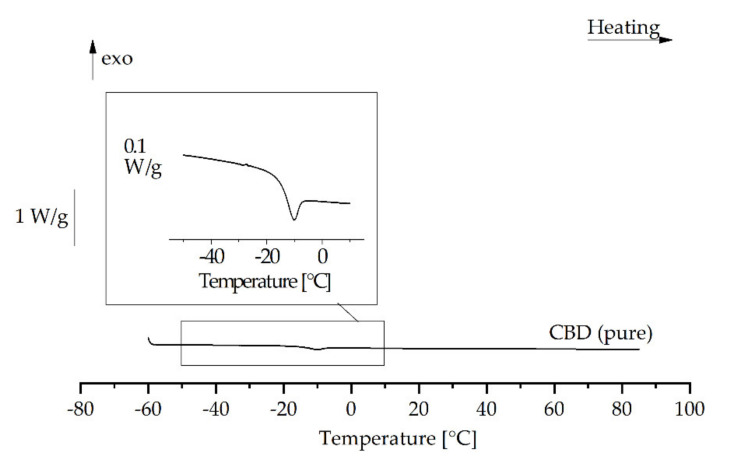
DSC reheating curve of cannabidiol (after heating to 85 °C and subsequent cooling to −60 °C).

DSC analysis of the CBD-loaded trimyristin emulsion indicated a strong decrease in the crystallization temperature of the lipid. Thus, in addition to the behavior of mixtures of CBD and trimyristin bulk materials (see Supplementary Information), the thermal behavior of CBD was also analyzed. A strong supercooling tendency of pure CBD was detected. When melted CBD was cooled to −60 °C, it seemed to solidify in an amorphous state, because no crystallization event could be observed and a glass transition was detected between −20 and −10 °C upon a subsequent reheating to 85 °C (Figure 7). The strong supercooling tendency may provide the possibility to produce colloidal emulsions with pure CBD as dispersed phase. The production of stable emulsions from a drug in the supercooled state has already been described for ubidecarenone [49].

**Figure 8:**
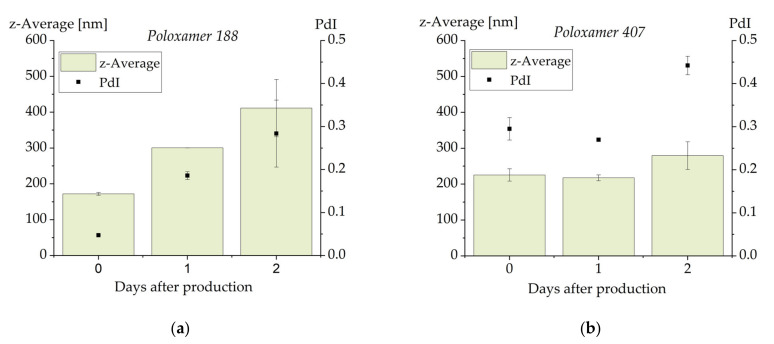
Particle size and PdI of emulsions containing pure cannabidiol as dispersed phase and stabilized with poloxamer 188 (**a**) or poloxamer 407 (**b**). The results represent the mean values of *n* = 2. The error bar shows the upper and lower measured values.

To produce emulsions of pure CBD with only a very small amount of material, dual centrifugation was applied as small-scale approach for emulsion preparation [50]. With this method, melt emulsification of CBD with Pol 188 as emulsifier led to the formation of a colloidal emulsion with a particle size below 200 nm (z-average) and a PdI of less than 0.05 (Figure 8a). Since comparable particle sizes were obtained in two centrifugation vials, these results seem to be reproducible. After one day of storage at room temperature, however, the particle size had increased to approximately 300 nm and the PdI into the range of 0.2. Two days after production, the considerable increase in particle size and PdI as well as the large error bars indicate that the emulsions had become unstable.

In a second approach, the emulsifier was changed to poloxamer 407. The particle size (above 200 nm) and PdI (about 0.3) achieved with this emulsifier were less favorable than with Pol 188 but remained unchanged one day after production. An increase in particle size and particularly in PdI was observed two days after production. DSC investigations on both formulations on all three days indicated that no sample contained crystalline material. The instability can thus not be explained by crystallization of CBD. Ostwald ripening can also be ruled out as the cause of instability since there was an increase in particle size as well as in PdI (Ostwald ripening should lead to a decrease in the PdI) [51]. An explanatory approach for the low stability of the CBD emulsions may be that the interaction of the poloxamer molecules with the molecules of CBD is less favorable than their interaction with triglycerides. This hypothesis is supported by the highly decreased stability of poloxamer-stabilized soybean oil emulsions in the shaking test after loading with high CBD concentrations.

Tendentially, the poloxamer 407-stabilized CBD emulsions appeared to be slightly more stable than the poloxamer 188-containing emulsions which on the other hand had smaller droplets and a narrower particle size distribution directly after production. Further formulation research may lead to CBD emulsions with further increased stability, potentially after an adaption of the formulation or inclusion of further processing steps. As a consequence of the stability assessments on CBD-loaded soybean oil emulsions and the published results of the ubidecarenone study [49], a change of the emulsifier to phospholipids and bile salts may be a possible improvement.

## 4. Conclusions

Colloidal lipid emulsions are a promising option for the formulation of CBD. The achievable CBD load was extraordinarily high for all investigated systems, whereby the absolute drug load depended on the lipid used and its physical state. Although the stability of the emulsions was limited under mechanical stress conditions, initial interventions demonstrated that there are ways to improve the stability.

Concerning the physicochemical properties of the lipid core, loading with CBD caused an increase in the density of the matrix which became less pronounced with increasing CBD concentration. In addition to its localization and interaction with the lipid matrix, CBD was partly localized at the interface of the emulsion droplets.

The strong supercooling tendency of CBD enabled the production of colloidal emulsions in which the dispersed phase only consisted of the drug. Despite the low stability of these emulsions of supercooled pure CBD, the results indicate a further interesting option in CBD formulation.

## Figures and Tables

**Table 1:** Structure and physicochemical properties of cannabidiol; green areas represent a lipophilic surface whereas grey areas are neutral and purple areas are hydrophilic.

pKa ^1^	Log P ^1^	Melting Point [°C] ^2^	Molar Mass [g/mol]
9.1	6.1	67	314
**Structure**		**Lipophilicity ^3^**	
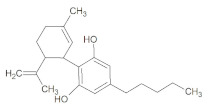		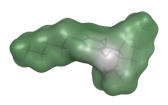	

^1^ [6]; ^2^ [17]; ^3^ simulated with MOE Version 2018.01.01.

**Table 2:** Composition of emulsions and process parameters. HPH = High-pressure homogenization; PME = premix membrane emulsification.

	Lipid	Emulsifier	Preservative	Process Parameters
*Kinetics of drug loading, influence of the lipid matrix and influence of drug load on emulsion stability*				
SB-P188-120 nm	10% Soybean oil	5% Poloxamer 188	0.05% Sodium azide	HPH 700 bar
TM-P188-120 nm	5% Trimyristin	6% Poloxamer 188	0.05% Sodium azide	HPH 500 bar
RS-P188-120 nm	10% Rapeseed oil	5% Poloxamer 188	0.05% Sodium azide	HPH 700 bar
MCT-P188-120 nm	10% Miglyol 812 (MCT)	5% Poloxamer 188	0.05% Sodium azide	HPH 700 bar
SB-P407-130 nm	10% Soybean oil	5% Poloxamer 407	0.05% Sodium azide	HPH 700 bar
*Drug localization*				
SB-P188-70 nm	10% Soybean oil	5% Poloxamer 188	0.05% Sodium azide	HPH 1500 bar
SB-P188-150 nm	10% Soybean oil	5% Poloxamer 188	0.05% Sodium azide	HPH 300 bar
SB-P188-230 nm	10% Soybean oil	5% Poloxamer 188	0.05% Sodium azide	PME 0.2 mm PE membrane

**Table 3:** Parameters applied for drug loading procedures.

Initially Added CBD Concentration	Sample Volume	Time of Drug Loading
*Kinetics of drug loading*		
30 mg/mL	1 mL	Equal to sample time
*Influence of the type of lipid matrix*		
30 mg/mL	1 mL	7 days
*Influence of drug load on emulsion stability*		
60 mg/mL	6 mL	7 days
*Drug localization*		
30 mg/mL	1 mL	14 days

**Table 4:** Particle size and emulsifier content in the aqueous phase of emulsions.

	PCS z-Average Diameter	PCS PdI	Emulsifier Content in the Aqueous Phase	Lipid Content after Production
*Kinetics of drug loading, influence of the type of lipid matrix and influence of drug load on emulsion stability*				
SB-P188-120 nm	122 nm	0.13	0.8%	n.d.
TM-P188-120 nm	119 nm	0.12	2.5%	n.d.
RS-P188-120 nm	124 nm	0.12	1.0%	n.d.
MCT-P188-120 nm	117 nm	0.11	2.0%	n.d.
SB-P407-130 nm	128 nm	0.11	0.2%	n.d.
*Drug localization*				
SB-P188-70 nm	69 nm	0.11	n.d.	8.9%
SB-P188-150 nm	151 nm	0.14	1.6%	8.6%
SB-P188-230 nm	233 nm	0.02	n.d.	7.9%

n.d. not determined; PCS: photon correlation spectroscopy.

**Table 5:** Oil composition according to the manufacturer’s certificate of analysis [38,39,40,41] and melting point of trimyristin [17].

Fatty Acids	Miglyol^®^ 812 (MCT)	Dynasan^®^ 114 (Trimyristin)	Soybean Oil	Rapeseed Oil
C8:0	55.8%	-	-	-
C10:0	43.3%	-	-	-
C12:0	0.6%	-	-	-
C14:0	-	100%	-	-
C16:0	-	-	10.4%	4.4%
C18:1	-	-	23.2%	64%
C18:2	-	-	53%	18.7%
C18:3	-	-	7.2%	8.1%
Melting points [°C]	Liquid at RT	57	Liquid at RT	Liquid at RT

RT: room temperature.

## Data Availability

Data is contained within the article or supplementary material.

## Supplementary Materials

The supplementary material (text file) contains further information on the correlation between CBD loading and the interfacial area of the emulsions (Supplement 1) as well as information on the thermal behavior of trimyristin-CBD bulk mixtures (including a description of the method used). The supplementary material is available online.

## Sample Availability

Samples of the lipid carrier systems are not available from the authors.
