# Supplementary material for: Formulation of Cannabidiol in Colloidal Lipid Carriers
Source: Molecules. 2021 Mar 8;26(5):1469. doi: 10.3390/molecules26051469 (PMC7962959; doi:10.3390/molecules26051469)
Supplement: Supplementary file 1 [file molecules-26-01469-s001.pdf]

Supplementary Information

for

Formulation of cannabidiol in colloidal lipid carriers

Nadine Monika Francke[1], Frederic Schneider[2], Knut Baumann[2,3], Heike Bunjes[1,3*]

[1] Technische Universität Braunschweig, Institut für Pharmazeutische Technologie und Biopharmazie, Mendelssohnstraße 1, 38106 Braunschweig, Germany

[2] Technische Universität Braunschweig, Institut für Medizinische und Pharmazeutische Chemie, Beethovenstraße 55, 38106 Braunschweig, Germany

[3] Technische Universität Braunschweig, Zentrum für Pharmaverfahrenstechnik (PVZ), Franz-Liszt-Straße 35A, 38106 Braunschweig, Germany

* Corresponding author, Tel: +49 531 391 5652; E-Mail: heike.bunjes@tu-braunschweig.de

Supplement 1: Correlation between drug load and interfacial area

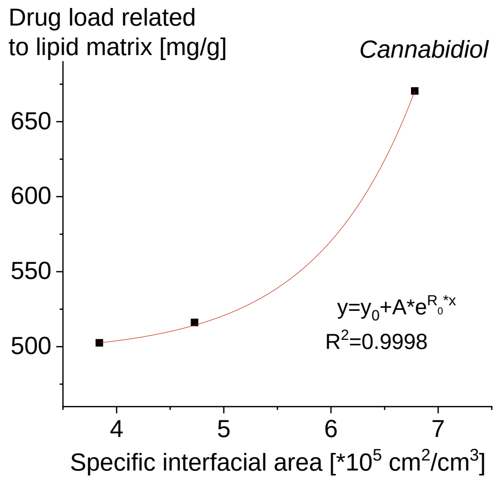

Figure S1: Correlation between achievable cannabidiol loading and available interfacial area of the emulsion droplets.

Supplement 2: Influence of cannabidiol on the thermal behavior of trimyristin

Methods:

The influence of CBD on the thermal behavior of trimyristin and mixtures of trimyristin-CBD-bulk material was analyzed. The DSC apparatus and applied crucibles were comparable to the method described in the main text of the article.

The two substances were weighed into the crucibles in defined ratios and slightly mixed with a metal needle. Two different temperature protocols were used with equal heating and cooling rates, but different temperature intervals. In the first protocol, the samples were subjected to the following temperature program: 1. Heating from 0 °C to 75 °C with a heating rate of 5 K/min; 2. Cooling from 75 °C to -15 °C with a cooling rate of 2.5 K/min; 3. Heating from -15 °C to 75 °C with a heating rate of 5K/min. The second protocol was performed in the following order: 1. Heating from 0 °C to 85 °C; 2. Cooling from 85 °C to -60 °C; 3. Heating from -60 °C to 85 °C.

Results:

Cooling of pure cannabidiol from the melt did not result in a crystallization peak even when cooling down to -15 °C (Figure S2A). This substance obviously displays pronounced supercooling. Under the same

conditions, trimyristin shows a single, clearly defined crystallization peak at 31 °C. An addition of increasing fractions of cannabidiol to trimyristin led to a flattening of the crystallization peak and to a linear reduction of the crystallization temperature (Figure S2B). This indicated eutectic behavior of the two substances as it is also known for the interaction of ubidecarenone with solid triglycerides [1].

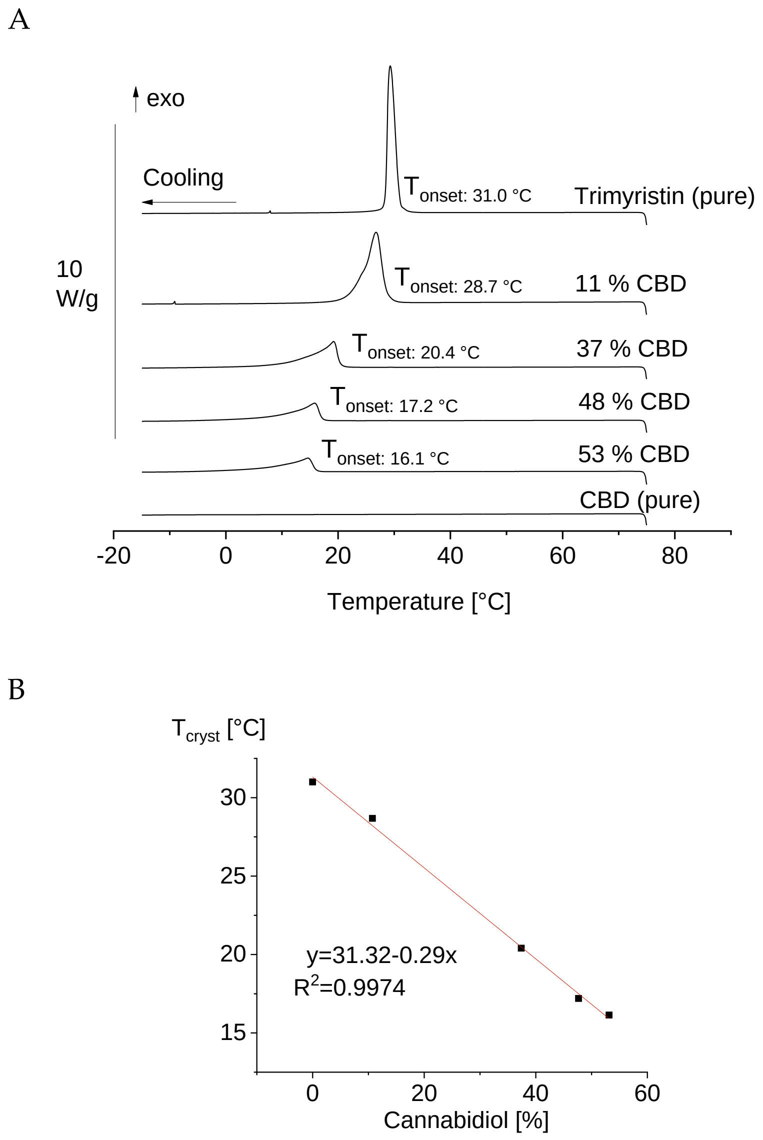

Figure S2: (A) DSC cooling curves of trimyristin, cannabidiol and their mixtures after heating to 75 °C; (B) Influence of cannabidiol on the crystallization temperature (T_{onset}) of triymristin. The cannabidiol fractions are given in mass percent.

Upon re-heating a very small endothermic event, two exothermic events and one large endothermic event (at 54 °C) were observed for trimyristin reflecting its polymorphic behavior [2,3]. The small melting events may indicate superimposed melting and recrystallization events of the metastable modifications finally leading to melting of the stable β modification (large event). An addition of 11 % cannabidiol (mass percent)

almost led to the disappearance of the small exothermic signals. Only one exothermic peak remained, which increased in intensity with increasing cannabidiol addition. This might be due to a transformation of the α-modification into the β-modification. According to the literature, the melting point of the α-modification of trimyristin is 33 °C and the β-modification melts at 56 °C [2]. The shift of the thermal events to lower temperatures can be explained by the eutectic behavior mentioned above.

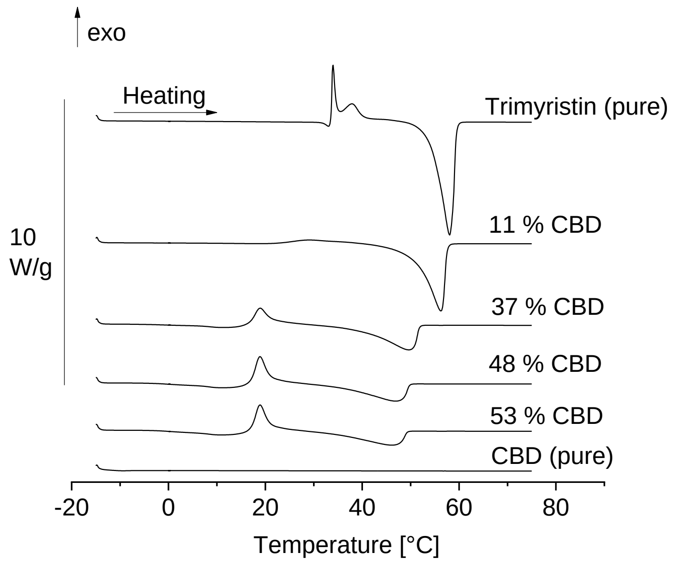

Figure S3: DSC re-heating curves of trimyristin, cannabidiol and mixtures (after heating to 75 °C and subsequent cooling to -15 °C)

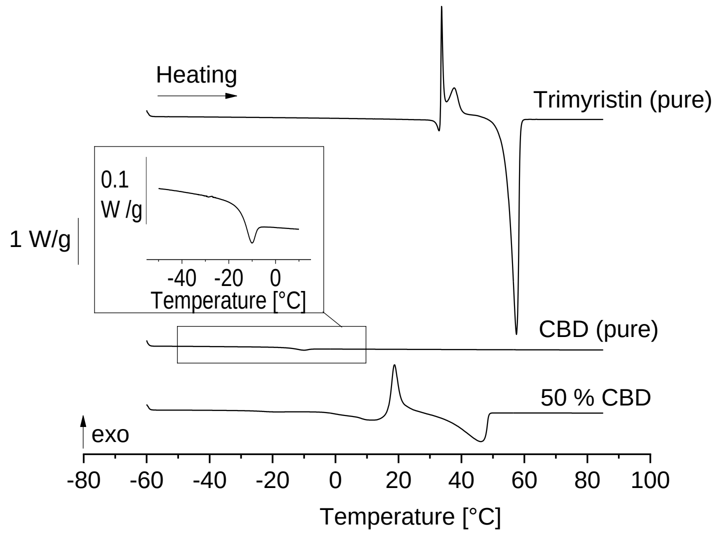

Figure S4: DSC re-heating curves of trimyristin, cannabidiol and a mixture (after heating to 85 °C and subsequent cooling to -60 °C)

The thermal behavior of trimyristin and a 50 % cannabidiol mixture upon re-heating after a preceding cooling step to -60 °C (Figure S4) was comparable to the results shown in Figure S3. For cannabidiol, a glass transition occurred between -20 °C and -10 °C (see main text), which was also detectable in the mixture of trimyristin with 50 % CBD.

References:

[1] H. Bunjes, M. Drechsler, M.H.J. Koch, K. Westesen, Incorporation of the model drug ubidecarenone into solid lipid nanoparticles, Pharm. Res. 18 (2001) 287–293. https://doi.org/10.1023/A:1011042627714.

[2] H. Bunjes, K. Westesen, M.H.J. Koch, Crystallization tendency and polymorphic transitions in triglyceride nanoparticles, Int. J. Pharm. 129 (1996) 159–173. https://doi.org/10.1016/0378-5173(95)04286-5.

[3] K. Sato, Polymorphism of lipid crystals, 1th ed., John Wiley & Sons, Ltd., Hoboken, 2018.
